# Interlinking the Cross Talk on Branched Chain Amino Acids, Water Soluble Vitamins and Adipokines in the Type 2 Diabetes Mellitus Etiology

**DOI:** 10.2174/0118715303305579241014112730

**Published:** 2025-01-08

**Authors:** P. Shruthi Rai, Yashodhar P. Bhandary, Y.M. Shivarajashankara, B. Akarsha, Roopa Bhandary, R.H. Prajna, K.G. Namrata, C.G. Savin, Priya Alva, P. Katyayani, Sukanya Shetty, T.J. Sudhakar

**Affiliations:** 1 Department of Biochemistry, KVG Medical College and Hospital, Sullia 574327, India;; 2 Yenepoya Research Centre, Yenepoya Deemed to be University, Mangalore, Karnataka 575018, India;; 3 Department of Biochemistry, K S Hegde Medical Academy, Mangalore, Karnataka 575018, India;; 4 Department of Microbiology, KVG Medical College and Hospital, Sullia 574327, India;; 5 Department of Pharmacology, KVG Medical College and Hospital, Sullia 574327, India;; 6 Department of Biochemistry, Tejasvini Institute of Allied Health Sciences, Mangalore, Karnataka 575025, India

**Keywords:** Branched-chain amino acids, diabetes mellitus, insulin resistance, vitamins, adipokines, mTOR

## Abstract

Type 2 Diabetes Mellitus (T2DM) is an etiologically diverse metabolic dysfunction that, if untreated, leads to chronic hyperglycemia. Understanding the etiology of T2DM is critical, as it represents one of the most formidable medical challenges of the twenty-first century. Traditionally, insulin resistance has been recognized as the primary risk factor and a well-known consequence of type 2 diabetes. Emerging evidence suggests that branched-chain amino acids (BCAAs), adipokines, and deficiencies in water-soluble vitamins, such as thiamine and pyridoxine, play significant roles in the development of insulin resistance, a key feature of T2DM. These factors are interconnected through the AMP-activated protein kinase (AMPK) pathway, which regulates various metabolic processes, including glucose transport, lipid synthesis, and inflammatory responses. Dysregulation of AMPK is linked to insulin resistance and metabolic syndrome-related illnesses. Understanding the interplay between BCAAs, adipokines, vitamins, and AMPK may offer new therapeutic targets for the prevention and treatment of diabetes mellitus.

## 1 INTRODUCTION

It is commonly known that type 2 Diabetes Mellitus (T2DM) is a deranged metabolic condition with a variety of etiologies that, in the absence of therapy, results in chronic hyperglycemia. It also includes issues with the metabolism of carbohydrates, fats, and proteins that are brought on by deficiencies in insulin secretion, insulin action, or both [1].

The global incidence of diabetes is alarmingly increasing, particularly in developing nations like India, owing to an increase in overweight/obesity and unhealthy lifestyles. T2DM is estimated to affect 77 million individuals in India as of 2019, and that number is expected to rise to around 134 million by the year 2045. About 57% of these individuals are still undiagnosed [2].

Understanding the etiology of T2DM is crucial for public health because it is unquestionably one of the most difficult health issues of the twenty-first century. The biggest risk factor was thought to be insulin resistance for T2DM and the well-known complications associated with it. In the etiology of diabetes mellitus, amino acids are increasingly recognized as a distinct class of potent molecules.

 Increased fasting levels of circulating branched-chain amino acids (BCAAs) have been linked to greater insulin resistance, according to epidemiological studies [3]. BCAAs play a crucial role in regulating glucose metabolism and protein synthesis. However, their potential impact on longevity is still under investigation and requires further research for conclusive evidence. Additionally, the relationship between BCAAs and insulin resistance is complex and contradictory. Studies have shown that elevated BCAA levels are linked to insulin resistance, diabetes, and metabolic complications like diabetic retinopathy and nephropathy [4-6]. However, elevated levels of circulating BCAA may be a sign of increasing insulin resistance and the result of reduced insulin action [7].

Thiamine and pyridoxine, key water-soluble vitamins, are involved in the metabolism of BCAAs. Thiamine and pyridoxine play crucial roles in glucose metabolism and insulin function, impacting insulin resistance [8, 9]. Thiamine deficiency can exacerbate diabetes complications by stimulating insulin resistance [10]. On the other hand, pyridoxine, or vitamin B6, affects lipid metabolism and adipogenesis, influencing insulin resistance through various pathways [9, 10].

Several studies have also shown that the levels of anti-inflammatory molecules like adiponectin are lowered in insulin-resistant stages of type 2 diabetes, even if inflammatory mediators or indicators such as IL-6, TNF-alpha, fibrinogen, high-sensitive CRP, and plasminogen activator inhibitor-1 (PAI-1) have increased [11, 12]. The most significant contributors to the development of insulin resistance are adiponectin, TNF-alpha, and IL-6, despite the fact that various other adipokines have been implicated in the pathogenesis of T2DM [13]. This review attempts to correlate plasma BCAA levels to adipokines, vitamins, and insulin resistance in the etiology of diabetes mellitus.

## 2 BRANCHED-CHAIN AMINO ACIDS AND INSULIN RESISTANCE

Branched-chain amino acids (BCAAs) are also recognized as significant dietary signals and may contribute to the etiology of type 2 diabetes mellitus and insulin resistance [14]. The quantities of BCAAs, such as valine, leucine, and isoleucine, change depending on the protein-rich meal that is consumed [4]. An earlier study found that individuals with significantly elevated levels of the amino acid valine had a 251% increased risk of developing diabetes compared to those with lower levels [15, 16].

The start and progression of type 2 diabetes can be predicted by BCAA plasma concentrations, according to several prospective studies with long-term follow-up [17, 18]. However, the majority of studies have concentrated on patients with obesity and/or poor glucose control; it is unclear how amino acids would affect people with early-stage glucose dysregulation.

BCAAs, which are considered key nutritional signals, may have a role in the etiology of insulin resistance and type 2 diabetes mellitus [14]. Race, gender, nutrition, and gene variations are key factors that determine BCAA levels and influence their connection with insulin resistance [19]. As per the study reported by earlier experts in these fields on various ethnic groups, Asian individuals (Chinese or Japanese) appeared to be more prone to bigger Homeostatic Model Assessment for Insulin Resistance (HOMA-IR) and higher BCAA levels than the Western population (Caucasians or Europeans) [20]. In terms of gender differences, most research has found that obese males have higher BCAA levels and a stronger positive correlation with insulin resistance compared to obese females. In summary, racial and gender differences can significantly impact BCAA levels and insulin resistance [21-25]. It has not yet been determined or researched how dietary habits affect BCAA levels and insulin resistance. The research findings have revealed that a diet rich in BCAA content may have a slightly favorable impact on peripheral BCAA levels [26]. This effect is most commonly explained by the 'insulinotropic' characteristics of amino acids or the lower glycemic load of high-protein diets [26].

BCAA has been proposed as a possible causal component in the insulin resistance (IR) etiology and T2DM by contributing to mitochondrial overload with lipid substrates, which leads to mitochondrial stress and decreased insulin action [27, 28]. Fig. (**1**) illustrates the mechanism of induction of insulin resistance due to excess accumulation of BCAA. Glucose and lipid metabolism are primarily regulated by the hormone insulin. Insulin that is released interacts with the insulin receptor under physiologically normal circumstances, encouraging the insulin receptor substrate (IRS)-1/2's tyrosine phosphorylation and controlling signals from the downstream cascade. This mechanism has been associated with the kinases PI3K, 3-phosphoinositide-dependent protein kinase (PDK), Akt, and S6K1. Serine phosphorylation of IRS proteins is a key component of the feedback control of insulin signalling. Tyrosine phosphorylation, ectopic glucose transporter synthesis, and translocation are all prevented by this process, which ultimately leads to IR [29]. Leucine, in particular, has been shown in several studies to enhance insulin signal transduction [30, 31]. Leucine enhanced the phosphorylation of Akt and mTOR in response to insulin and increased IRS-1 tyrosine phosphorylation, inhibiting insulin resistance induced by high-fat diet in insulin-target organs [32]. The rise in GLUT4 levels, glucose absorption, and insulin signal transduction could have been brought on by energetic expenditure that was accompanied by protein synthesis brought on by BCAAs or leucine [33-35].

Leucine, however, may interfere with insulin signal transduction *via* additional processes. Leucine at high concentrations inhibited AMPK activation while simultaneously boosting mTOR/p70S6K signalling and causing IR in rat skeletal muscle [36]. Leucine deprivation may also enhance hepatic insulin sensitivity by successively activating the amino acid sensor general control non-derepressible (GCN) 2 and reducing mTOR/p70S6K signaling [37].

Plasma levels of branched-chain amino acids (BCAAs) valine, leucine, and isoleucine have been the focus of attention among amino acids, owing to their role in skeletal muscle energy metabolism and the fact that leucine has been demonstrated to promote protein synthesis *in vitro* [38]. BCAA levels in the blood have been proven to predict nutritional status in hemodialysis patients [39].

Further research is needed to determine the precise mechanisms by which BCAAs regulate insulin sensitivity and glucose metabolism under various conditions.

## 3 VITAMINS AND INSULIN RESISTANCE

Vitamins are necessary micronutrients that biological organisms must have in small amounts to maintain normal cellular metabolism. Thiamine and pyridoxine are important water-soluble vitamins involved in the breakdown of BCAAs. Fig. (**2**) represents the role of thiamine and pyridoxine in the BCAAs metabolism.

Thiamine, also known as vitamin B1, was the first vitamin to be discovered [40]. The decarboxylation of BCAAs and alpha-keto acids serves as a catalyst for the production of energy. Thiamine, in the form of thiamine pyrophosphate, also serves as a coenzyme for transketolase reactions.

Despite a few human pilot studies demonstrating the advantages of thiamine administration in managing diabetic nephropathy, its potential role in delaying the development or progression of diabetic retinopathy has not yet been investigated [41-43]. Diabetes is thought to be a state of low thiamine due to increased glucose metabolism [41]. The earlier researchers concluded that plasma thiamine levels are decreased and their excretion increased in type 2 diabetes mellitus and also found positive dyslipidemia with thiamine deficiency [44, 45]. A study supported the link between blood thiamine levels and T2DM patients with early-stage renal failure [46]. Pyridoxine serves as a cofactor in the initial step of the degradation of BCAAs by branched-chain aminotransferase. One of the studies concluded that rats given a diet enriched with branched-chain amino acids while suffering from vitamin B6 deficiency had a high incidence of fat deposition in their liver [47]. Pyridoxine has also been demonstrated to lessen insulin resistance in some trials [48-50]. Leucine and vitamin B6 work together synergistically to diminish adipocyte lipid storage while increasing insulin sensitivity, muscular fat oxidation, and oxidative and inflammatory stress [50]. Pyridoxine has also been demonstrated in several trials to lower fasting blood sugar levels [48, 51].

## 4 ADIPOKINES AND INSULIN RESISTANCE

Adipose tissue is currently regarded as a highly specialized tissue that performs a vital endocrine function by producing and secreting a variety of bioactive chemicals such as adiponectin, leptin, resistin, and cytokines such as tumor necrosis factor-alpha (TNF-α) and interleukin-6 (IL-6) [11, 12]. Insulin governs anti-lipolytic processes and has a wide range of impacts on metabolic processes in adipocytes, therefore, worsening of cell sensitivity to this hormone or impairment of the insulin pathway may alter adipose tissue metabolism. Insulin resistance is connected to increased TNF-α expression, and IL-6 plays a key role in lipid buildup in the myocardium [13].

Fig. (**3**) explains the role of both anti-inflammatory and pro-inflammatory cytokines in the etiology of insulin resistance. Hyperadiposity, metabolic syndrome, and T2DM are known to affect adipokines such as leptin and adiponectin. While both adipokines are produced by adipose tissue, obese people have higher amounts of leptin and lower levels of adiponectin [52, 53]. Adipocyte differentiation is aided by leptin and adiponectin concentration [54-56]. In addition, the correlation between the ratio of leptin to adiponectin and systemic inflammation, as well as its ability to forecast insulin resistance among non-diabetic individuals, highlights the involvement of adipose tissue malfunction in the development of insulin resistance [57, 58].

Numerous studies have investigated alterations in the levels of these adipocytokines in individuals with confirmed diabetes or those undergoing antidiabetic drug therapy. However, there is a dearth of research on how genetic predisposition affects plasma levels and the possible contribution to the onset and progression of insulin resistance and diabetes in individuals. TNF-α and IL-6, adipocyte-derived inflammatory adipokines, were found to be associated with insulin resistance, whereas adiponectin, an anti-inflammatory adipokine, was not associated [59].

### 4.1 Connecting The Dots of BCAA, Vitamins, Adipokines and Insulin Resistance

The interconnection of BCAAs, vitamins, adipokines, and insulin resistance is mediated by the AMP-activated protein kinase (AMPK). Initially identified as an enzyme capable of enhancing cellular ATP production (such as fatty acid oxidation) and reducing ATP consumption for biochemical processes (such as fatty acid, triglyceride and protein synthesis), AMPK is activated by changes in the AMP/ATP ratio, resulting in an increase in cellular ATP concentration [60]. AMPK plays a regulatory role in numerous physiological processes such as cellular growth and proliferation, mitochondrial function and biogenesis, and insulin resistance- related factors, including inflammation, oxidative and endoplasmic reticulum stress, and autophagy, in addition to glucose transport, lipid and protein synthesis, and fuel metabolism [61]. In humans, AMPK dysregulation plays a key role in the pathophysiology of insulin resistance and metabolic syndrome-related illnesses. This cross-sectional investigation will strongly demonstrate that AMPK dysregulation and IR are linked. Finally, it appears that the discovery of effective and specific AMPK activators is on the horizon [61]. If this is the case, we may be able to assess the therapeutic efficacy of AMPK activation in the prevention and treatment of diabetes mellitus in the near future. Fig. (**4**) illustrates the possible mechanism that involves BCAAs, adipokines, vitamins, and AMPK in the pathogenesis of insulin resistance.

Branched-chain amino acids, notably leucine, have been shown to promote mTOR signaling, protein synthesis, and insulin resistance [62]. BCAAs have been reported to inhibit AMPK activation while enhancing mTOR/p70S6K signaling and protein synthesis in recent studies [63]. Moreover, when human adipocytes are stimulated by inflammatory and/or metabolic stimuli, AMPK regulates adipokine expression and secretion profiles [64]. Insulin resistance is triggered by an imbalance in the adipokine profile [63]. In addition, the activity of AMPK is regulated by adipokines, which can impede the pro-inflammatory and pro-proliferative actions of adipokines such as TNF-α, IL-6, IL-8, and leptin while enhancing the anti-inflammatory and anti-proliferative effects of adipokines such as adiponectin and IL1-RA in insulin-resistant tissues [63]. Adiponectin enhances fatty acid oxidation (AMP kinase activation), resulting in a drop in plasma FFA levels but an increase in glucose consumption [65].

Indeed, Liu *et al.* demonstrated that adiponectin restored the altered BCAA metabolism caused by high-fat diets in mice, while Lian *et al.* discovered that deficits in adiponectin signaling were linked to a reduction in BCAA catabolism enzyme activity [66, 67]. The earlier researchers discovered a positive relationship between plasma BCAA and leptin levels, as well as an inverse relationship between BCAA and adiponectin, including high molecular weight (HMW) adiponectin levels [68].

In experimental animals, adipokines such as TNF-α, IL-6, Retinal binding protein-4 (RBP4), IL-18, lipocalin 2, as well as BCAA levels are linked to insulin resistance, although there is little evidence in humans [69-73]. Some studies report that there is a positive association between vitamins and the development of insulin resistance [46-50].

BCAAs, in particular, may affect some adipocytes, controlling the adipokines that promote insulin resistance. Moreover, due to the absence of a definitive correlation, the potential link between BCAA levels and adipokines may be attributable to residual confounding. The deficiency of vitamins like thiamine and pyridoxine may result in increased plasma BCAA levels and, initiate the activation of mTOR/P70S6k and induce the development of insulin resistance.

## 5 DISCUSSION

The incidence of insulin resistance, T2DM, and metabolic syndrome is escalating at alarming rates worldwide. It is generally known that chronic nutritional overabundance causes insulin resistance. The development of diabetes mellitus is considerably influenced by insulin resistance, which stems from abnormalities in insulin-mediated glucose uptake and/or glucose release in peripheral tissues, liver, and muscles.

Essential amino acids, including leucine, isoleucine, and valine, which are classified as BCAAs, impact metabolism either directly or indirectly [74-76]. They are found in quite substantial levels in dietary proteins, accounting for 15-20% of protein intake. The elevated levels of metabolic byproducts of BCAAs and mitochondrial oxidation of glucose and lipids, both of which generate mitochondrial stress [77].

Water soluble vitamins, such as thiamine and pyridoxine, act as cofactors in BCAA catabolism; deficiency of these vitamins leads to elevation in plasma BCAA levels.

Metabolic dysfunction of BCAAs and sustained activation of mTORC1 may underlie the association between BCAAs and the risk of metabolic disorders, including insulin resistance and T2DM, regardless of body mass index (BMI). This suggests that BCAAs may serve as an early predictor of these conditions [74]. The expression of branched-chain amino acid catabolism genes in visceral adipose tissue is linked to insulin sensitivity. When obese people with metabolic syndrome are compared to obese people without metabolic syndrome, this gene expression is found to be lower [74]. Reduced metabolism of BCAA, which is influenced by adipose tissue [74, 78], may contribute to the association between hyper adiposity and insulin resistance.

AMPK is an enzyme that senses energy levels and plays a role in nutrition sensing and insulin sensitivity. It is a heterotrimeric protein that signals to increase ATP-generating processes and decrease ATP-consuming processes during periods of low energy.

A decrease in AMPK activity and phosphorylation of AMPK Thr172 seems to be an initial and fundamental step towards insulin resistance induced by excessive dietary intake. According to Viollet *et al.,* AMPK activity can be reduced by a variety of hormones and inflammatory cytokines in addition to nutrition [78]. Furthermore, because AMPK downregulation is a typical occurrence in response to all of these nutritional stimuli, its stimulation is an obvious therapeutic target. Diet, exercise, and insulin-sensitizing pharmaceutical treatments like metformin, all of which activate AMPK, are being used to treat T2DM and the metabolic syndrome [18]. While these treatments are beneficial, they are frequently insufficient to control patients' blood glucose levels on their own, necessitating the development of more powerful drugs.

Decreased AMPK activation is caused by mechanisms that raise plasma BCAA levels, vitamin insufficiency, and an increase in pro-inflammatory cytokines. The detection of markers that indicate the transition from physiological to pathological insulin resistance, which is associated with other detrimental processes like inflammation and oxidative stress, could be useful in identifying potential targets. Enhancing our understanding of AMPK regulation and the factors that contribute to its downregulation could lead to the discovery of novel chemical AMPK activators, as well as other molecules in the pathway that could be targeted in conjunction with AMPK activators to enhance insulin resistance alleviation.

## CONCLUSION

In conclusion, elevated levels of BCAAs, pro-inflammatory adipokines, and deficiencies in vitamins can significantly impact cellular metabolism by disrupting mitochondrial function and promoting inflammatory responses. These disruptions contribute to the development of insulin resistance, further exacerbating metabolic disorders. Further research is essential to determine whether abnormal concentrations of BCAAs, vitamin deficiencies, and adipokines could serve as reliable biomarkers or therapeutic targets for the clinical diagnosis and treatment of diabetes mellitus. Additionally, it is crucial to explore whether these factors collectively influence energy metabolism and inflammation through other signaling pathways, which may reveal new avenues for intervention.

## Figures and Tables

**Fig. (1):**
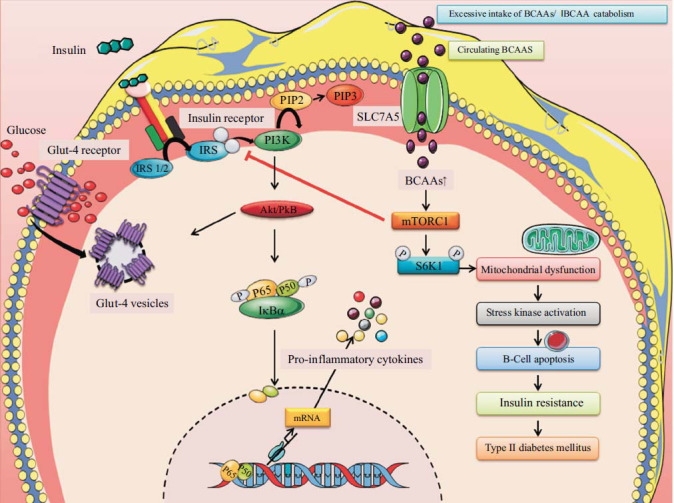
In this schematic diagram, the activation of the mTORC1 (mammalian target of rapamycin complex 1) in response to branched-chain amino acids (BCAAs) and its impact on insulin resistance are depicted. Insulin receptor substrate 1 (IRS-1) is inhibited by mTORC1 and S6K1 after being activated by BCAAs at serine residues 307, 636/639, 1101, and 312. Affected Akt/protein kinase B activity through negative feedback regulation reduces insulin responses and activates NF-KB, which causes the production of cytokines that promote inflammation. S6K1phosphorylation induces mitochondrial dysfunction and induction of stress kinase, resulting in apoptosis of β-cell of the pancreas and insulin resistance. “The Figure was created using Servier Medical Art, licenced under a Creative Commons Attribution 3.0 unported licence (https://smart.servier.com (Accessed 17 April 2023))”.

**Fig. (2):**
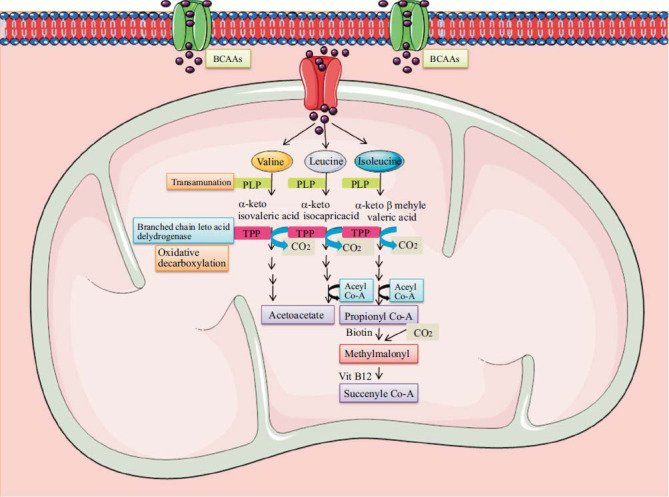
Diagrammatic representation of the degradation of branched-chain amino acids. Branched-chain aminotransferase enzyme (BCAT) catalyses the first step in the breakdown of BCAA and needs PLP to function as a cofactor. Thiamine pyrophosphate (TPP), a coenzyme, is required for the E1 component of the branched chain alpha-keto acid dehydrogenase complex (BCKDH) in the subsequent step. Through a sequence of enzyme reactions, metabolites of BCAAs come together to generate the final product and enter into the Tricarboxylic acid cycle. Lack of pyridoxine and thiamine causes an increase in hazardous branched-chain amino acid metabolites as well as BCAA, which can cause mitochondrial malfunction and beta-cell death. The multi-step reaction is indicated by the dotted line. “The Figure was created using Servier Medical Art, licenced under a Creative Commons Attribution 3.0 unported licence (https://smart.servier.com (Accessed on 17 April 2023)”.

**Fig. (3):**
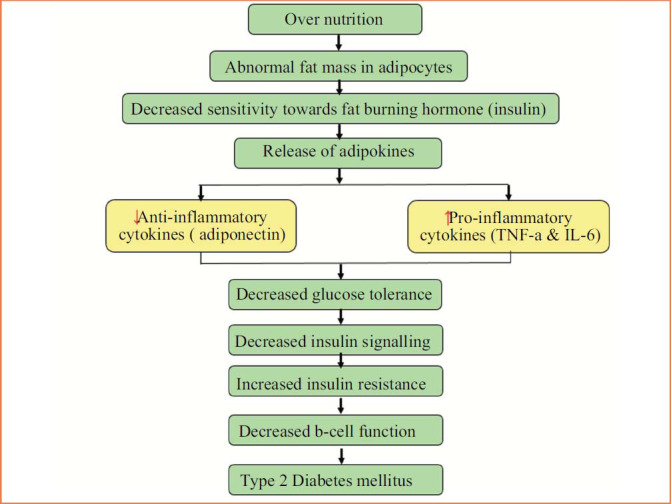
Flow chart illustrating the involvement of adipokines in the progression of insulin resistance that includes tumor necrosis factor-alpha (TNF-α) and Interleukin 6 (IL-6).

**Fig. (4):**
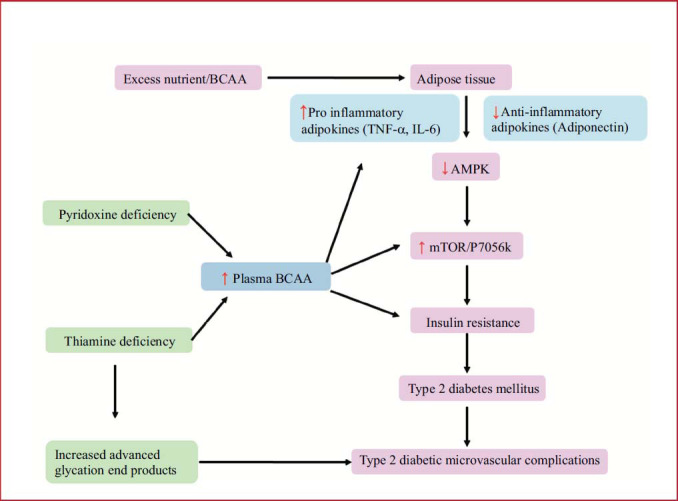
The Flow chart elucidates the possible mechanisms of insulin resistance involving BCAAs, adipokines, vitamins, and AMPK.

## LIST OF ABBREVIATIONS

AMPK: AMP-Activated Protein Kinase
BCAAs: Branched-Chain Amino Acids
BMI: Body Mass Index
IR: Rnsulin Resistance
